# The U-Shaped Relationship Between Serum Uric Acid and Long-Term All-Cause Mortality in Coronary Artery Disease Patients: A Cohort Study of 33,034 Patients

**DOI:** 10.3389/fcvm.2022.858889

**Published:** 2022-06-22

**Authors:** Yiying Zheng, Jiaman Ou, Dehua Huang, Ziyou Zhou, Xiaoli Dong, Jie Chen, Dandan Liang, Jin Liu, Yong Liu, Jiyan Chen, Xiaoyu Huang, Ning Tan

**Affiliations:** ^1^The Second School of Clinical Medicine, Southern Medical University, Guangzhou, China; ^2^Department of Cardiology, Guangdong Cardiovascular Institute, Guangdong Provincial People's Hospital, Guangdong Academy of Medical Sciences, Guangzhou, China; ^3^Guangdong Provincial Key Laboratory of Coronary Heart Disease Prevention, Guangdong Cardiovascular Institute, Guangdong Provincial People's Hospital, Guangdong Academy of Medical Sciences, Guangzhou, China; ^4^Department of Cardiology, People's Hospital of Yangjiang, Yangjiang, China; ^5^Guangdong Provincial People's Hospital, School of Medicine, South China University of Technology, Guangzhou, China; ^6^Department of Ultrasound Imaging, Yunnan Fuwai Cardiovascular Hospital, Kunming, China; ^7^Department of Cardiology, Guangdong Medical University, Zhanjiang, China

**Keywords:** serum uric acid, all-cause mortality, coronary artery disease, U-shape, cardiovascular diseases

## Abstract

**Background:**

Associations between high serum uric acid (SUA) and cardiovascular diseases have been reported. However, few studies have been conducted to explore the relationship between SUA and long-term all-cause mortality in coronary artery disease (CAD) patients. Our study aims to investigate the relationship between SUA and long-term all-cause mortality in patients with CAD.

**Methods:**

From January 2007 to December 2018, we divided 33,034 patients with CAD admitted in the Guangdong Provincial People's Hospital into five groups (quintile 1: SUA <5.05 mg/dl, quintile 2: 5.05 mg/dl ≤ SUA <5.59 mg/dl, quintile 3:5.59 mg/dl ≤ SUA <6.8 mg/dl, quintile 4, 6.8 mg/dl ≤ SUA <7.93 mg/dl, and quintile 5, SUA ≥7.93 mg/d;). This study used Kaplan–Meier survival analysis to evaluate patient outcomes with different ranges of SUA. Cox proportional hazards regression models and restricted cubic spline were applied to determine the association between serum uric and long-term all-cause mortality.

**Results:**

A total of 33,034 participants were recruited, including 24,780 (75.01%) men and 8,254 (24.99) women in this cohort study. Median follow-up was 4.91 years. We found that SUA is an independent risk factor of long-term all-cause mortality according to the result of Cox proportional hazards models. This study also illustrated an approximate U-shape association between SUA and all-cause mortality when compared with 5.95 mg/lL ≤ SUA <6.8 mg/dl, SUA <5.0 5mg/dl (adjusted hazard ratio (aHR) =1.13, 95% *CI*: 1.01–1.26, *p* = 0.03), and SUA ≥8 mg/dL (aHR = 1.18, 95% *CI*: 1.06-1.32, *p* = 0.003).

**Conclusion:**

Our study indicated a U-shaped relationship between SUA and long-term all-cause mortality in patients with CAD. No matter whether SUA is too high or too low, it increased the all-cause mortality in the CAD population, which deserves to be closely monitored.

## Introduction

The Global Burden of Diseases Study showed that the number of cases of coronary artery diseases (CAD) doubled from 271 million in 1990 to 523 million in 2019, and the incidence of the number of CAD deaths reached 18.6 million globally, accounting for 38% of all global burden of cardiovascular diseases in 2019. There were 10,636,000 new cases were estimated worldwide in 2017 alone (1–3). It is well established that a great variety of potential outcomes are related to CAD, such as major adverse cardiovascular events, heart failure, admission, physical disabilities, and reduced activities of daily living (1). As the end oxidation product of purine metabolism in humans (4, 5), serum uric acid (SUA) has received increased attention for the association with cardiovascular disease and renal disease (6). Some studies suggested that high SUA level is an independent risk factor for CAD, renal disease, and all-cause mortality (6–8).

Still, some studies demonstrated that even a low SUA level has high mortality considering that a too low SUA level may indicate other comorbidities (9, 10). Further, a U-shaped relationship between SUA and all-cause mortality was found in the general population and patients with high atherosclerotic risk (11, 12).

Among general patients with CAD, some previous studies suggested that SUA is linearly related to prognosis (7). Some other studies categorized serum SUA into several strata to explore the association (11).

The definite relation between SUA and prognosis in general patients with CAD is still unknown. For the patients with CAD, the optimal control range of SUA is not clear. Therefore, this study is intended to assess the association between baseline SUA and long-term all-cause mortality in patients with CAD based on a real-world cohort.

## Methods

### Study Design and Populations

**Figure 1:**
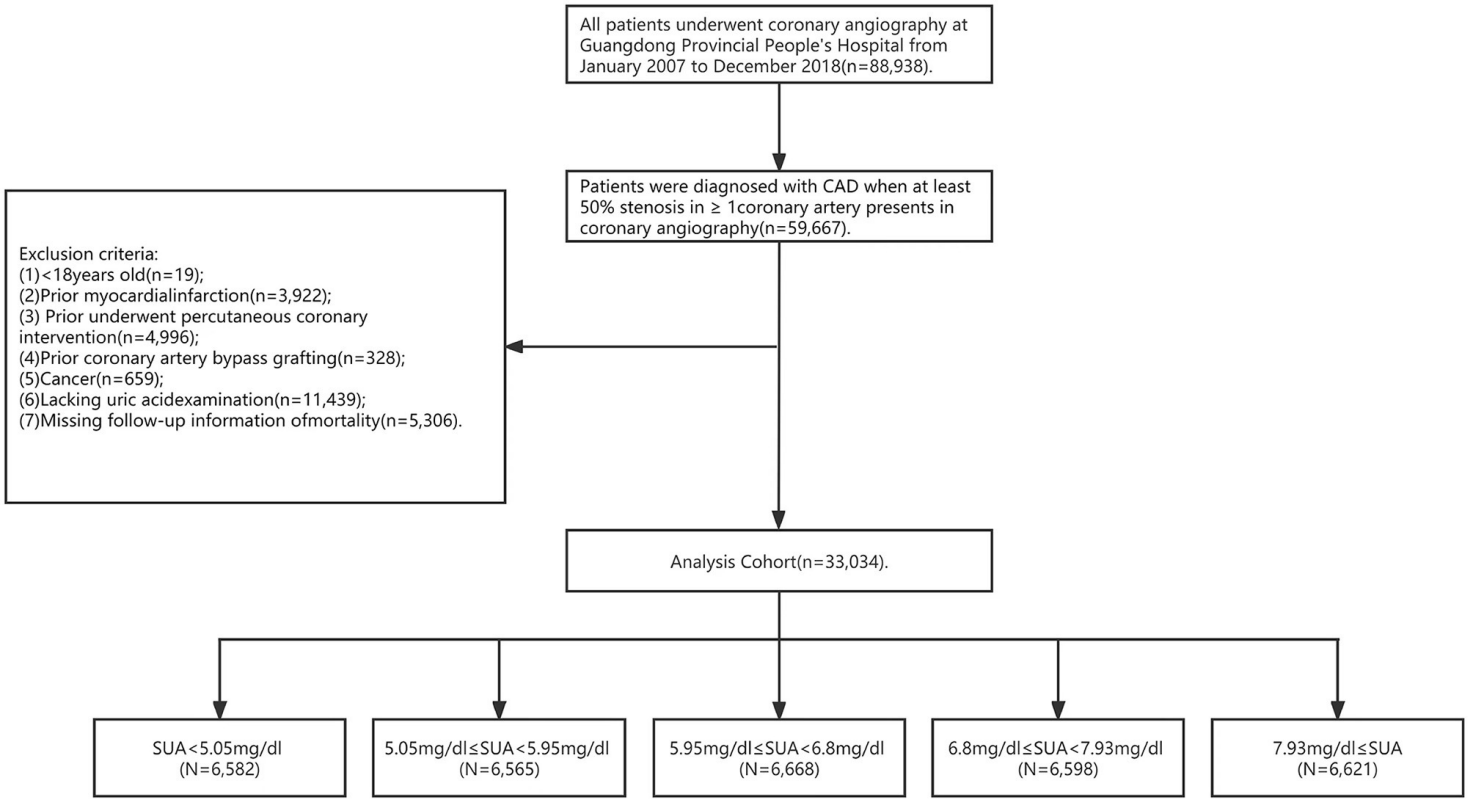
Study flow chart.

This study was based on data of the Cardiorenal ImprovemeNt (CIN) study (Clinicaltrials.gov NCT04407936). A total of 88,938 consecutive patients who underwent coronary angiography between January 2007 and December 2018 in the Guangdong Provincial People's Hospital were included. The detailed inclusion and exclusion criteria of the current study are shown in Figure 1. The exclusion criteri of the current study were as follows: (i) <18 years old (*n* = 19); (ii) prior myocardial infarction (*n* = 3,922); (iii) prior percutaneous coronary intervention (*n* = 4,996); (iv) prior coronary artery bypass grafting (*n* = 328); (v) cancer *(n* = 659); (vi) missing SUA values (*n* = 11.439); and (vii) missing follow-up information of mortality (*n* = 5.306). Baseline clinical data including demographic characteristics, coexisting conditions, nutritional status, laboratory tests, and medicine at discharge were collected. All data were extracted from the Electronic Clinical Management System of Guangdong Provincial People's Hospital. The Ethics Committee of Guangdong Provincial People's Hospital authorized this study, which was in compliance with the Declaration of Helsinki.

### UA Measurement

Uric acid was measured once at admission before PCI, and venous blood samples were drawn in the morning following an overnight fast. The serum sample was separated and analyzed with an analyzer using a colorimetric procedure. Uric acid was determined by the enzymatic uricase method. All measurements were taken with blinded quality control specimens in the laboratory. A serum UA level of >7 mg/dl in men and >6 mg/dl in women was defined as hyperuricemia (13).

### Endpoint and Definition

The endpoint of the study was defined as long-term all-cause death. CKD was defined according to the Kidney Disease: Improving Global Outcomes Organization (KDIGO) clinical practice guideline, in which eGFR <60 ml/min/1.73 m2 was determined as CKD (14). According to the World Health Organization criteria, the criteria for anemia in adult men and adult women are hemoglobin <120 g/L and <110 g/, respectively. Patients were diagnosed with CAD when at least 50% stenosis in ≥1 coronary artery presents in coronary angiography. Other comorbidities such as diabetes mellitus, hypertensions, etc., were defined using the International Classification of Diseases, 10th revision diagnostic codes.

### Statistical Analysis

Enrolled patients were grouped into five categories according to the quintiles of SUA (quintile 1: SUA <5.05 mg/dl, quintile 2: 5.05 mg/dl ≤ SUA <5.59 mg/dl, quintile 3:5.59 mg/dl ≤ SUA <6.8 mg/dl, quintile 4, 6.8 mg/dl ≤ SUA <7.93 mg/dl, quintile 5, SUA ≥7.93 mg/dl). The characteristics of age, uric acid, HCT, lymphocyte, neutrophil, total cholesterol, HDL-C, LDL-C, triglyceride, and ALB in Table 1 were continuous; we used normal distribution. For the other characteristics in Table 1, we adopted describable percentages. All normal distribution continuous variables were summarized using means and standard deviation, and were compared using the *t*-test. Non-normal distribution continuous variables were summarized using median and interquartile range, and were compared using the variance (ANOVA). Categorical variables were measured in percentages or absolute numbers and compared using the Pearson chi-squared test.

**Table 1:** Baseline characteristics.

Characteristic*	**Overall** **(*n* = 33,034)**	**SUA <5.05 mg/dL** **(*n* = 6,582)**	**5.05 ≤SUA <5.59 mg/dL** **(*n* = 6,565)**	**5.59 ≤SUA <6.8 mg/dL** **(*n* = 6,668)**	**6.8≤SUA <7.93 mg/dL** **(*n* = 6,598)**	**SUA ≥7.93 mg/dL** **(*n* = 6,621)**	***P*-value**
**Demographic characteristics**							
Age, year	63.09 (10.59)	63.33 (10.10)	62.83 (10.40)	62.69 (10.40)	62.87 (10.78)	63.72 (11.21)	<0.001
Age ≥75 years, *n* (%)	4,941 (14.96)	948 (14.40)	914 (13.92)	881 (13.21)	977 (14.81)	1221 (18.44)	<0.001
Male, *n* (%)	24,780 (75.01)	4,040 (61.38)	4,759 (72.49)	5,177 (77.64)	5,413 (82.04)	5,391 (81.42)	<0.001
**Coexisting conditions**							
PCI, *n* (%)	24,277 (73.49)	4,932 (74.93)	4,891 (74.50)	4,940 (74.09)	4,813 (72.95)	4,701 (71.00)	<0.001
AMI, *n* (%)	7,161 (21.68)	1,822 (27.68)	1,489 (22.68)	1,292 (19.38)	1,244 (18.85)	1,314 (19.85)	<0.001
CHF, *n* (%)	3,248 (9.84)	543 (8.25)	459 (7.00)	494 (7.41)	655 (9.94)	1,097 (16.58)	<0.001
Hypertension, *n* (%)	18,754 (56.77)	3,436 (52.20)	3,476 (52.95)	3,704 (55.55)	3,888 (58.93)	4,250 (64.19)	<0.001
Diabetes mellitus, *n* (%)	9,122 (27.61)	2,122 (32.24)	1,766 (26.90)	1,717 (25.75)	1,698 (25.74)	1,819 (27.47)	<0.001
CKD, *n* (%)	5977(18.09)	516 (7.73)	657 (9.96)	894 (13.44)	1,362 (20.25)	2,584 (39.37)	<0.001
Atrial fibrillation, *n* (%)	1,070 (3.24)	145 (2.17)	141 (2.14)	188 (2.83)	227 (3.47)	369 (5.62)	<0.001
COPD, *n* (%)	289 (0.87)	47 (0.71)	52 (0.79)	47 (0.70)	71 (1.08)	72 (1.09)	0.02
Stroke, *n* (%)	1,993 (6.03)	407 (6.18)	362 (5.51)	364 (5.46)	417 (6.32)	443 (6.69)	0.01
Anemia, *n* (%)	10,487 (32.12)	2,393 (36.82)	1,990 (30.73)	1867 (28.36)	1929 (29.57)	2,308 (35.14)	<0.001
**Laboratory examination**							
Serum uric acid, mg/dL	6.56 (1.86)	4.28 (6.18)	5.53(0.26)	6.37 (0.24)	7.32 (0.32)	9.36 (1.43)	<0.001
HGB, g/l	133.19 (16.93)	129.92 (15.75)	133.49 (15.63)	134.96 (16.03)	135.02 (16.87)	132.58 (19.53)	<0.001
HCT	0.40 (0.37,0.43)	0.39 (0.36,0.42)	0.40 (0.37,0.43)	0.41 (0.38,0.43)	0.41 (0.37,0.44)	0.40 (0.37,0.44)	<0.001
Lymphocyte, 10^9^/L	1.86 (1.45,2.34)	1.77 (1.36,2.24)	1.86 (1.47,2.33)	1.90 (1.48,2.38)	1.91 (1.48,2.40)	1.86 (1.43,2.37)	<0.001
Neutrophil, 10^9^/L	5.26 (2.63)	5.30 (2.73)	5.16 (2.52)	5.10 (2.46)	5.17 (2.54)	5.55 (2.86)	<0.001
Total cholesterol, mmol/L	4.59 (1.21)	4.51 (1.17)	4.58 (1.19)	4.61 (1.22)	4.63 (1.21)	4.64 (1.26)	<0.001
HDL-C, mmol/L	1.00 (0.26)	1.05 (0.29)	1.02 (0.26)	1.00 (0.26)	0.98 (0.24)	0.95 (0.24)	<0.001
LDL-C, mmol/L	2.86 (0.98)	2.75 (0.94)	2.84 (0.96)	2.87 (0.98)	2.90 (0.98)	2.93 (1.00)	<0.001
Triglyceride, mmol/L	1.39 (1.02,1.94)	1.24 (0.92,1.69)	1.31 (0.98,1.81)	1.39 (1.03,1.95)	1.48 (1.08,2.05)	1.56 (1.13,2.20)	<0.001
ALB, g/L	36.39 (4.26)	35.53 (4.56)	36.42 (4.06)	36.69 (4.01)	36.88 (4.09)	36.44 (4.44)	<0.001
**Medicine**							
RASi, *n* (%)	15,580 (48.02)	3,298 (50.92)	3,197 (49.32)	3,212 (48.93)	3,064 (47.14)	2,809 (43.75)	<0.001
β-blocker, *n* (%)	25,916 (79.88)	5,127 (79.16)	5,234 (80.75)	5,303 (80.78)	5,162 (79.42)	5,090 (79.28)	0.03
Statins, *n* (%)	30,650 (94.47)	6,096 (94.12)	6,181 (95.36)	6,287 (95.77)	6,150 (94.62)	5,936 (92.46)	<0.001
Hydrochlorothiazide, *n* (%)	1,272 (3.92)	200 (3.09)	226 (3.49)	238 (3.63)	283 (4.35)	325 (5.06)	<0.001
Furosemide, *n* (%)	4,379 (13.50)	575 (8.88)	632 (9.75)	704 (10.72)	883 (13.58)	1,585 (24.69)	<0.001
**Events**							
All-cause mortality, *n* (%)	3,936 (11.91)	816 (12.40)	674 (10.27)	706 (10.59)	733 (11.11)	1007 (15.21)	<0.001

*Data are presented as the mean value (standard deviation), median [interquartile range] or number of participants (percentage).

*PCI, percutaneous coronary intervention; AMI, acute myocardial infarction; CHF, congestive heart failure; CKD, chronic kidney disease; COPD, chronic obstructive pulmonary disease; HDL-C, high-density lipoprotein cholesterol; LDL-C, low-density lipoprotein cholesterol; pro-BNP, pro-brain natriuretic peptide; RASi, renin angiotensin system inhibitor*.

We applied Kaplan–Meier methods to analyze the prognosis, and Cox proportional hazards analyses to test the relationship between SUA and death from all causes in general patients of CAD. We used restricted cubic splines to explore the shape of the relation between them. Model 1 was unadjusted, model 2 was adjusted for age and sex, and model 3 was adjusted for age, sex, PCI, comorbidities such as AMI, CHF, hypertension, diabetes mellitus, CKD, anemia, atrial fibrillation, COPD, and stroke, medicine such as hydrochlorothiazide and, furosemide and uric acid-lowering drugs (allopurinol, febuxostat, and benzbromarone), and laboratory examination such as HDL-C, LDL-C, CKMB, and AST. We conducted subgroup analysis based on patients' characteristics and comorbidities stratified by age, sex, PCI, comorbidities such as AMI, CHF, hypertension, diabetes mellitus, CKD, anemia, atrial fibrillation, COPD and stroke, medicine such as hydrochlorothiazide and furosemide, and laboratory examination such as HDL-C and LDL-C.

All data analyses were performed using R software (version 3.6.3). We regarded a two-tailed *p*-value < 0.05 as statistically significant.

### Follow-Up Data and Clinical Results

Follow-up information cams from a comprehensive database of medical records, and information on the time and causes of cardiovascular death or coronary heart disease cams directly from medical records. The endpoint of the study was defined as long-term all-cause death. The National Death Registry Database registers valid information according to the 10th edition of the international classification of diseases (ICD-10), and the ICD-10 code of cardiovascular disease death is 390–459.

## Result

### Clinical Characteristics

A total of 33,034 participants were recruited, including 24,780 (75.01%) men and 8,254 (24.99) women in this cohort study. Baseline characteristics of this cohort study are shown in Table 1. The mean age was 63.09 ± 10.59 years. The average value of SUA for the 33,034 subjects was 6.56 ± 1.86 mg/dL, and patients were classified SUA into five groups. Overall, 24,277 (73.49%) patients with PCI, 18,754 (56.77%) patients with hypertension, 9,578 (29.35%) patients with diabetes mellitus, 5977 (18.09%) patients with CKD, 10,487 (32.12%) patients with anemia, 1,993 (6.03%) patients with stroke, 1,070 (3.24%) patients with atrial fibrillation, and 289 (0.87%) patients with COPD were in this cohort. Patients with hyperuricemia were more likely to be men and have a history of hypertension, CKD, and heart failure than those with normal uricemia (Table 1).

### Main Outcomes

**Figure 2:**
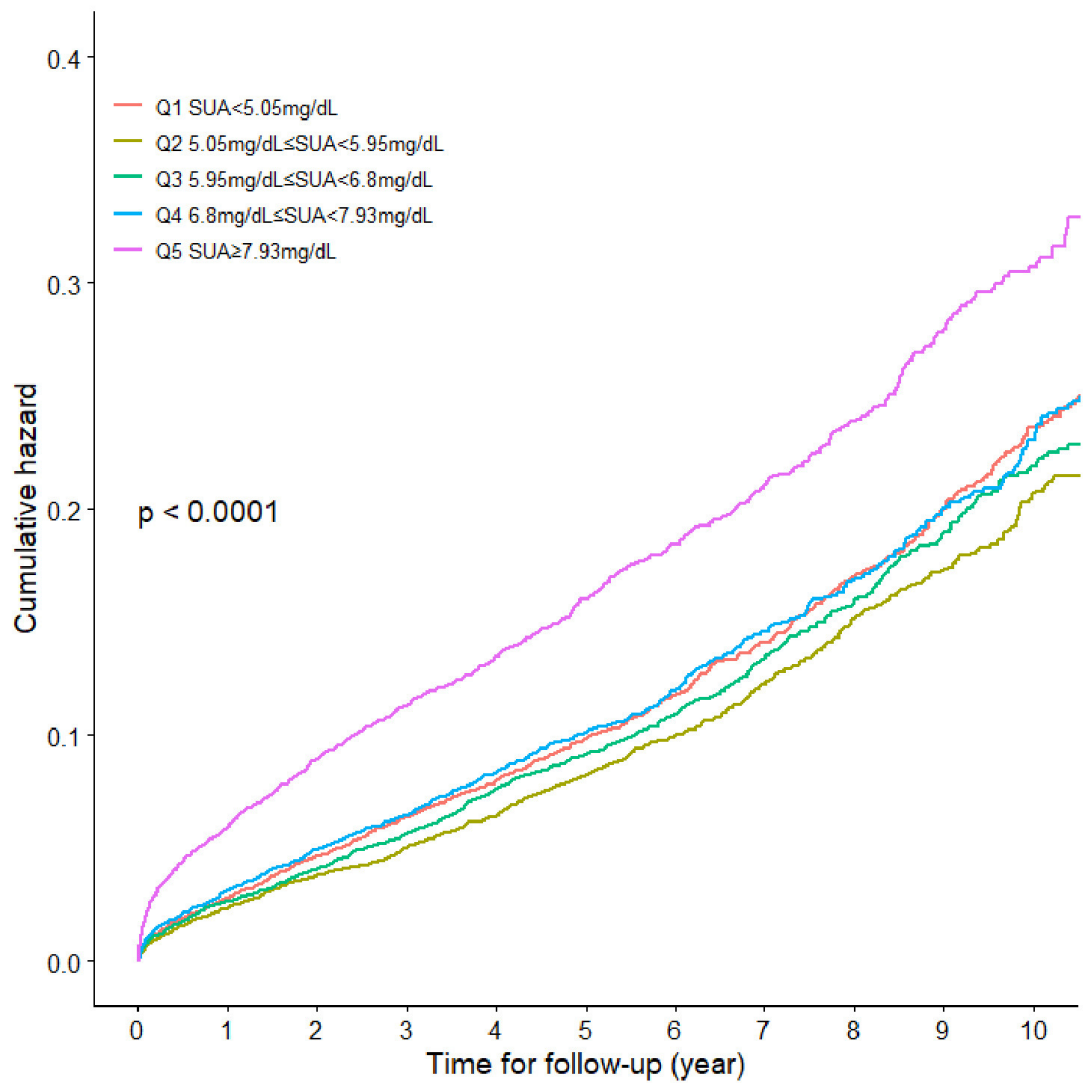
Kaplan-Meier curves for cumulative hazard of long-term all-cause mortality.

**Figure 3:**
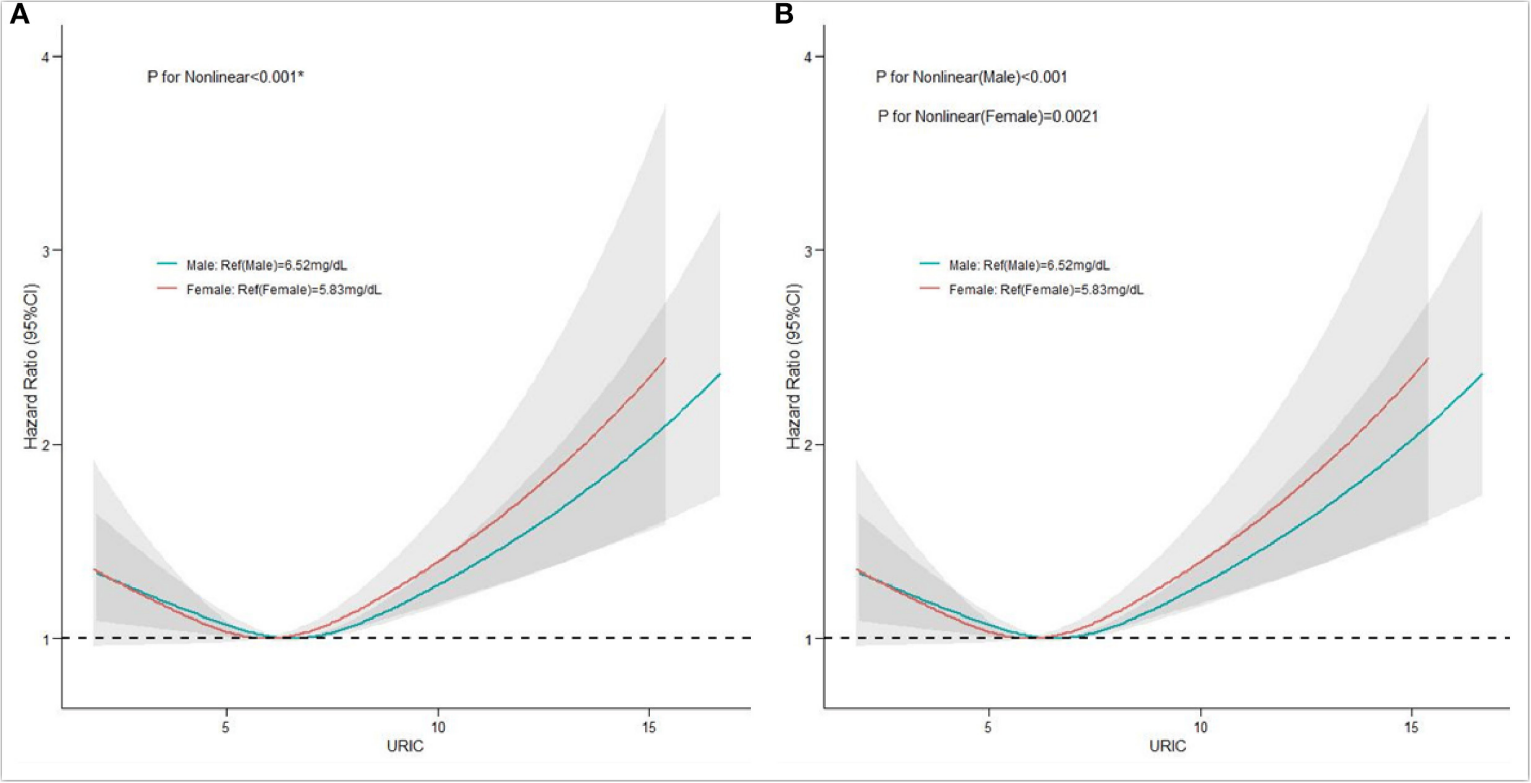
Restricted spline curve of the association between serum uric acid (SUA) level and hazard ratios (HRs) for all-cause mortality according to gender. **(A)** The restricted spline curve of the univariate Cox model; **(B)** the restricted spline curve of the multivariate Cox model.

**Figure 4:**
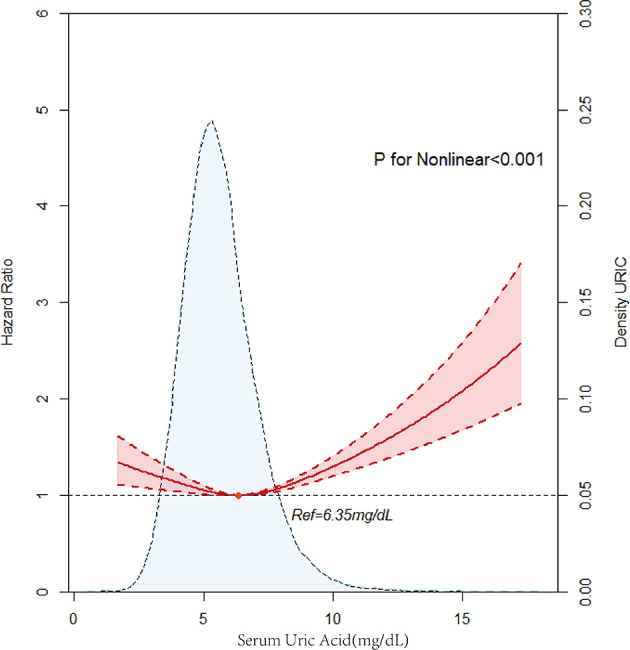
Multivariable adjusted cubic spline model for the association between serum uric acid (SUA) level and hazard ratios (HRs) for all-cause mortality.

A total of 3,936 patients died during the 4.91 (IQR 2.90–7.42) years of follow-up. The men were classified into the hyperuricemia group (SUA ≥7 mg/dl) and the hypouricemia group (SUA ≤ 3mg/dl). The women were classified into the hyperuricemia group (SUA ≥6 mg/dl), and the hypouricemia group (SUA ≤ 2.5 mg /dl). After adjustment for confounders, the hyperuricemia group had a higher risk of long-term mortality compared to the hypouricemia group in both men and women and was more significant in women (male hyperuricemia group, aHR = 0.84, 95% *CI*: 0.52–1.38, *p* = 0.500, male hypouricemia group, aHR = 1.03, 95% *CI*: 0.95–1.12, *p* = 0.443; female hyperuricemia group; aHR = 0.67, 95% *CI*: 0.22–2.1, *p* = 0.496, female hyperuricemia group, aHR = 1.00, 95% *CI*: 0.86–1.16, *p* = 0.984). Divided into five groups (quartiles 1, 2, 3, 4, 5), Kaplan–Meier curves of survival showed the highest all-cause mortality for the SUA >7.93 mg/dl subgroups; the SUA 5.05–5.95mg/dl subgroup had the least mortality (*p* < 0.0001) (Figure 2). After multivariable adjustment, Cox regression models using quintile 3 as the reference demonstrated that subjects in the lowest (quintile 1) or the highest SUA level group (quintile 5) had a significantly higher risk of long-term mortality compared to other groups [compared with quintile 3, quintile 1, adjusted hazard ratio (aHR) = 1.13, 95% *CI*: 1.01–1.26, *p* = 0.03; quintile 2, aHR = 0.96, 95%*CI*: 0.86–1.07, *p* = 0.471; quintile 4, aHR = 1.01, 95% *CI*: 0.91–1.13, *p* = 0.848; quintile 5, aHR = 1.18, 95%*CI*: 1.06–1.32, *p* = 0.003]. It was revealed that all-cause mortality was 13.0, −4.0, 1.0, and 18.0% in the SUA (0–5.05), SUA (5.05–5.95), SUA (5.95–6.80), and SUA >7.9 mg/dl subgroups. The results of Cox regression are shown in detail in Table 2. As depicted in Figure 3, after multivariable adjustment, cubic spline models showed a U-shaped association between SUA level with all-cause mortality among men and women, respectively. We found evidence of nonlinear associations between gender and all-cause mortality (*p* for nonlinear associations <0.001). The plot showed a substantial reduction of the risk within the lower range of predicted all-cause death, which reached the lowest risk at around 6.52 mg/dl in men (*p* for non-linearity <0.001) and 5.83 mg/dl in women (*p* for non-linearity = 0.0021) and then increased rapidly afterward. The significant non-linear relationship between SUA and all-cause mortality is revealed in Figure 4 (*p* < 0.001). Actually, Figure 4 visually confirmed a U-shaped relationship between SUA and all-cause mortality.

**Table 2:** Cox regression models for SUA and all-cause mortality.

	**Univariate cox regression**			**Multivariate cox regression**					
	**Model 1**			**Model 2**			**Model 3**		
	**HR**	**95% *CI***	***p*-value**	**HR**	**95% *CI***	***p*-value**	**HR**	**95% *CI***	***p*-Value**
**Continuous variable**									
SUA (per 1.67 mg/dL)	1.22	1.19–1.25	<0.001	1.19	1.16–1.23	<0.001	1.06	1.03–1.10	<0.001
**Categorical variable**									
Q1	1.09	0.99–1.21	0.093	1.11	1.01–1.23	0.049	1.12	1.01–1.26	0.038
Q2	0.93	0.84–1.03	0.172	0.93	0.84–1.04	0.206	0.97	0.86–1.08	0.541
Q3	Reference			Reference			Reference		
Q4	1.10	0.99–1.22	0.080	1.07	0.97–1.19	0.183	1.00	0.89–1.12	0.996
Q5	1.67	1.52–1.84	<0.001	1.59	1.44–1.75	<0.001	1.17	1.05–1.30	0.005

*Model 1, Unadjusted model*.

*Model 2, Adjusted for age and sex*.

*Model 3, Adjusted for all covariates: age, sex, PCI, comorbidities such as AMI, CHF, hypertension, diabetes mellitus, CKD, anemia, atrial fibrillation, COPD, and stroke, medicine such as drug hyperuricemia hydrochlorothiazide and furosemide, and laboratory examination such as HDL-CAST, CKMB, and LDL-C*.

### Subgroup Analysis

**Figure 5:**
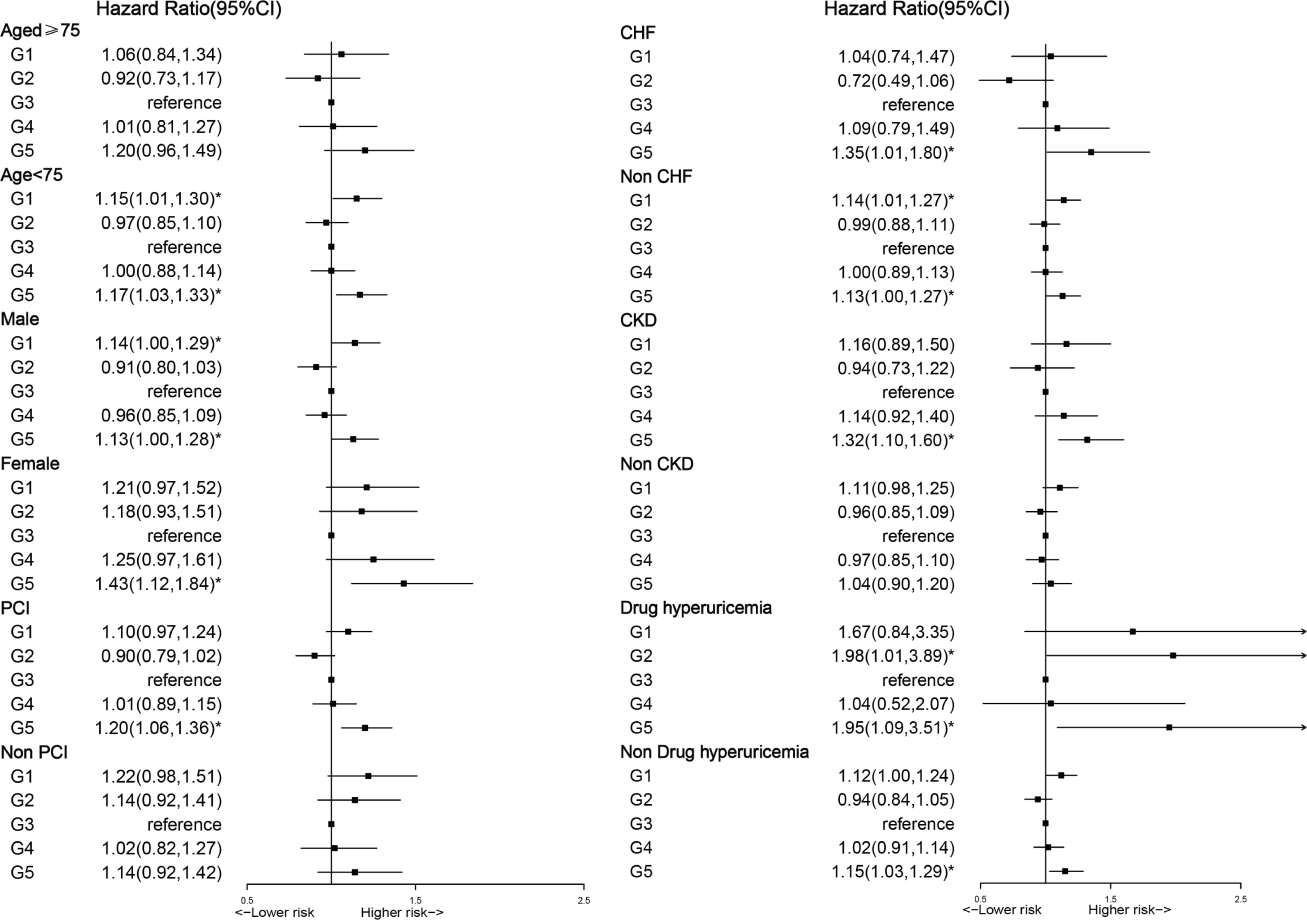
Cox proportional hazard ratios for long-term all-cause mortality in different subgroups.

A subgroup analysis was performed to explore whether the relationship between SUA and long-term mortality could be explained by patients' baseline characteristics and coexisting conditions, such as age, gender, PCI, CHF CKD, and uric acid-lowering drugs (such as allopurinol, febuxostat, and benzbromarone). The U-shaped relationship between SUA and long-term all-cause mortality was affected by uric acid-lowering drugs, but high levels of SUA were still associated with increased long-term all-cause mortality in the general patients with CAD (Figure 5). In subgroup analysis, the U-shaped relationship between SUA and all-cause mortality was more significant in the group without uric acid-lowering drugs than in the group taking uric acid-lowering drugs. With every 1 mg/dl increase in SUA of quintile 5, the risk of all-cause mortality increased by 15% (aHR = 1.15, 95% *CI*:1.03–1.29, *p* = 0.012), which was significantly lower than quintile 5 in the uric acid-lowering group (aHR = 1.95, 95% *CI*:1.09–3.51, *p* = 0.025).

## Discussion

To our knowledge, this cohort study is the first to access the relationship between SUA and long-term all-cause mortality in general patients with CAD, which revealed that low or high levels of SUA were independently associated with increased mortality. This observational study demonstrated a significant U-shaped association between SUA levels and all-cause mortality with a large cohort of 33,034 participants who had CAD.

Increased SUA concentration was established as an identified risk factor. Many findings showed that increased SUA levels predicted increased risk of all-cause mortality (15–17). Meanwhile, some studies demonstrated that low SUA levels were associated with increased risk of all-cause mortality (9, 10). A U-shaped association was also found in general patients and patients with high atherosclerosis risk. In an observational study conducted by Kuo et al., the findings showed a significant U-shaped association between SUA levels and adverse outcomes such as all-cause and cardiovascular mortality in 354,110 patients without a history of gout (11). Participants with SUA levels at either extreme are at higher risk for all-cause and cardiovascular mortality. In Tseng's longitudinal cohort including 127,771 adults aged 65 and older in Taiwan, the results demonstrated a U-shaped association between SUA levels and all-cause and cardiovascular disease mortality in older adults. When SUA levels were below 4 mg/dl or above 8 mg/dl, the risk of all-cause death and CVD-related death was also higher (18). Another observational study conducted by Cang et al. enrolled 3,047 patients and illustrated that both hyperuricemia and hypouricemia increased the risk of all-cause mortality. These studies are consistent with our finding of a U-shaped relationship between SUA levels and mortality in the CAD patient population.

Our study shows a U-shaped relationship, and the possible mechanisms are as follows: SUA can penetrate endothelial and muscle cells and intracellular hyperuricemia can produce inflammatory stress through ROS/active nitrogen (RNS) production and cyclooxygenase 2 activation. Oxidative stress and the activation of the renin-angiotensin system in human vascular endothelial cells is the main mechanism of SUA-induced endothelial dysfunction (19). These processes are involved in the occurrence and development of atherosclerosis, increasing the risk of CAD and other cardiovascular events. SUA impaired basic and VEGF-induced nitric oxide production in cultured endothelial cells (20). Nitric oxide levels are known to play a key role in promoting atherosclerosis and acute vascular events (21). Elevated SUA may increase mortality associated with thromboembolic disease. Several possible reasons could explain the association between high SUA and an increased risk of death. SUA plays an important role in immune regulation and tumor suppression (22). Thus, the increased mortality from hypouricemia is partly due to the incidence of cancer (23, 24). SUA is an antioxidant, can interact with hydrogen peroxide and hydroxyl radicals, and effectively removes free radicals, thus protecting vascular endothelial cells. Low SUA levels result in the loss of radical scavenging ability which may increase vascular endothelial cell damage, and thus increase the higher cardiovascular mortality (25, 26).

The results of this study indicate that both hyperuricemia and hypouricemia increased the all-cause mortality in the CAD population. In an analysis according to subgroup, the results showed that a U-shaped relationship between SUA and long-term all-cause mortality was found in patients <75 years, men, non-CHF, CKD, and using uric acid-lowering drugs (such as allopurinol, febuxostat, and benzbromarone). The U-shaped relationship between SUA and long-term all-cause mortality was affected by uric acid-lowering drugs. There are some studies that demonstrated that uric acid-lowering drugs are associated with an increased risk of cardiovascular events (27). And we found that in subgroup analysis, the use of uric acid-lowering drugs did not reduce all-cause mortality. According to existing research, it suggests that uric acid-lowering drugs can also cause cardiovascular death. Therefore, the benefit of uric acid lowering on asymptomatic hyperuricemia patients in the ASCVD population is unclear (28). Thus, we recommend that SUA, a easy to obtain hematology-based test, should be closely monitored in clinical practice, especially in the population of CAD. Clinicians are supposed to find the underlying cause of abnormal SUA by more in-depth clinical history, physical examination, and laboratory data. For low SUA levels of patients with CAD, monitoring of SUA is required. Regarding high SUA levels of patients with CAD, we strongly recommend using uric acid-lowering drugs and lifestyle modification interventions, such as regular exercise and dietary management, to lower SUA.

## Limitations

Our study had some limitations. First, as an observational single-center study, it limits our ability to give direct causal inferences. However, with a large sample size and long follow-up, the current study still performs well in representing patients with CAD in southern China. Second, SUA was gathered only at baseline yet the SUA may have changed over time. Nevertheless, it is more representative with unaffected SUA, which was collected at admission.

## Conclusion

This study demonstrated a U-shaped relationship between SUA and long-term all-cause mortality in general patients with CAD. Patients with CAD with an optimal SUA level (5.59 mg/dl ≤ SUA <6.8 mg/dl may have a better prognosis. The necessity of close monitoring of SUA is determined. A more active intervention to maintain optimal SUA control is probably warranted, which needs further intervention studies.

## Data Availability Statement

The raw data supporting the conclusions of this article will be made available by the authors, without undue reservation.

## Ethics Statement

The studies involving human participants were reviewed and approved by the Ethics Committee of Guangdong Provincial People's Hospital. Written informed consent for participation was not required for this study in accordance with the national legislation and the institutional requirements.

## Author Contributions

JL and YL had full access to data and take responsibility for the integrity, and the accuracy of the data analysis. YZ, JO, DH, ZZ, and XD concept and design. XD data management. YZ, JO, DH, ZZ, XD, JieC, DL, and JL drafting of the manuscript. YL, JiyC, and NT critical revision. NT final approval to publish. All authors contributed to acquisition, analysis, and interpretation data. All authors contributed to the article and approved the submitted version.

## Funding

This study was supported by Guangdong Provincial Science and Technology Project (2020B1111170011 and KJ022021049) and Guangdong Provincial Key Laboratory of Coronary Heart Disease Prevention (No. Y0120220151).

## Conflict of Interest

The authors declare that the research was conducted in the absence of any commercial or financial relationships that could be construed as a potential conflict of interest.

## Publisher's Note

All claims expressed in this article are solely those of the authors and do not necessarily represent those of their affiliated organizations, or those of the publisher, the editors and the reviewers. Any product that may be evaluated in this article, or claim that may be made by its manufacturer, is not guaranteed or endorsed by the publisher.
